# Nutritional status and risk factors of overweight and obesity for children aged 9–15 years in Chengdu, Southwest China

**DOI:** 10.1186/1471-2458-12-636

**Published:** 2012-08-10

**Authors:** Ping Li, Fan Yang, Fei Xiong, Tingzhu Huo, Yu Tong, Sufei Yang, Meng Mao

**Affiliations:** 1 Department of Pediatrics, West China Second University Hospital, Sichuan University, No. 17, People`s South Road, Chengdu, 610041, Sichuan Province, PR China; 2 Laboratory of Early Developmental and Injuries, West China Institute of Woman and Children’s Health, West China Second University Hospital, Sichuan University, Chengdu, PR China; 3 Key Laboratory of Obstetric & Gynecologic and Pediatric Diseases and Birth Defects of Ministry of Education, Chengdu, PR China; 4 Chengdu Women's and Children's Central Hospital, No. 1617, Riyue Avenue, Chengdu, 610091, Sichuan Province, PR China

## Abstract

**Background:**

Obesity is widespread in the world including developing countries. However malnutrition in poor areas is still a serious problem. Few investigations, especially in a large sample, have been performed in Western area of China. This study aimed to evaluate the nutritional status of school children aged 9–15 years in large Southwest city of China, and identify the differential impact of aberrant birth categories and family history of obesity related disease on childhood overweight and obesity development.

**Methods:**

A multistage random cluster sampling was performed to evaluate the prevalence of thinness, overweight and obesity, which were defined by the new age-, sex-, specific BMI reference developed by World Health Organization (WHO) (2007). And then a frequency matched case–control study was performed to identify the risk factors of overweight and obesity.

**Results:**

7,194 children (3,494 boys, 3,700 girls) were recruited, and 1,282 (17.8%) had excess bodyweight (14.5% overweight, 3.3% obesity). The combined prevalence gradually decreased with age, and were more prevalent among boys than girls (*P* <0.05). Meanwhile 6.3% were found thinness and there were little differences in genders (*P* >0.05). Preterm large for gestational age (OR = 2.746), maternal history of obesity related disease (OR = 1.713), paternal history of obesity related disease (OR = 1.583), preterm appropriate for gestational age (OR = 1.564), full term small for gestational age (OR = 1.454) and full term large for gestational age (OR = 1.418) were recognized as significant risk factors in the multivariate regression analysis (*P* <0.05).

**Conclusions:**

While overweight and obesity was dramatically spreading, malnutrition still remained a serious problem. This unmatched nutritional status should be emphasized in backward cities of China. Children born of both preterm and LGA, whose parents particularly mothers had a history of obesity related disease, should be emphatically intervened as early as possible.

## Background

Overweight and obesity has become mounting prevalent worldwide, even among children in developing countries [1]. Most of the wildspread chronic non-communicable diseases (CNCDs) such as type 2 diabetes (T2DM), primary hypertension and coronary heart disease (CHD) have been considered being strongly associatied with obesity [2,3]. Moreover it must be noted that adulthood obesity and its related disease are usually trace back to obesity in childhood [4-6]. Therefore, overweight and obesity in childhood should be effectively intervened as early as possible.

**Table 1:** Prevalence of thinness, overweight and obesity at different ages in boys and girls according to the new reference of WHO (2007)

	**Age (years)**	**No.**	**Thinness,% (no.)**	**Overweight,% (no.)**	**Obese, % (no.)**	**Excess body weight, % (no.)**
Boys	9-10	400	6.5 (26)	22.5 (90)	12.8 (51)	35.3 (141)
	10-11	683	6.4 (44)	22.4 (153)	8.2 (56)	30.6 (209)
	11-12	595	4.5 (27)	23.7 (141)	5.5 (33)	29.2 (174)
	12-13	728	5.9 (43)	24.2 (176)	3.8 (28)	28.0 (204)
	13-14	772	6.2 (48)	17.5 (135)	3.1 (24)	20.6 (159)
	14-15	316	8.5 (27)	13.9 (44)	2.2 (7)	16.1 (51)
		3,494	6.2 (215)^n.s.^	21.2 (739)*	5.7 (199)*	26.8 (938)*
Girls	9-10	462	5.6 (26)	11.3 (52)	2.2 (10)	13.4 (62)
	10-11	800	7.1 (57)	11.8 (94)	1.0 (8)	12.8 (102)
	11-12	613	6.2 (38)	6.7 (41)	1.5 (9)	8.2 (50)
	12-13	806	6.3 (51)	7.3 (59)	0.5 (4)	7.8 (63)
	13-14	767	7.3 (56)	6.6 (51)	0.8 (6)	7.4 (57)
	14-15	252	4.0 (10)	3.2 (8)	0.8 (2)	4.0 (10)
		3,700	6.4 (238)^n.s.^	8.2 (305)*	1.1 (39)*	9.3 (344)*
Boys& girls	9-10	862	6.0 (52)	16.5 (142)	7.1 (61)	23.5 (203)
	10-11	1,483	6.8 (101)	16.7 (247)	4.3 (64)	21.0 (311)
	11-12	1,208	5.4 (65)	15.1 (182)	3.5 (42)	18.5 (224)
	12-13	1,534	6.1 (94)	15.3 (235)	2.1 (32)	17.4 (267)
	13-14	1,539	6.8 (104)	12.1 (186)	1.9 (30)	14.0 (216)
	14-15	568	6.5 (37)	9.2 (52)	1.6 (9)	10.7 (61)
Total		7,194	6.3 (453)	14.5 (1,044)	3.3 (238)	17.8 (1,282)

Note. χ^2^ test; *P <0.05, statistically significant difference; n.s.: not significant.

**Table 2:** Comparisons of parental income, living space, birth categories and family history of ORD between cases and controls

**Variables**		**Cases (n = 1,282),% or median with 95% CI**	**Controls (n = 2,586),% or median with 95% CI**	**χ**^**2**^	** *P* **
Parental income (RMB/year)^A^	< 20,000	11.7	10.0	7.903	0.095
	20,000-50,000	25.2	25.3		
	50,000-100,000	34.9	38.4		
	100,000-20,000	19.7	19.4		
	≥ 200,000	8.5	6.9		
Living space (m^2^)^B^		110.3 (107.7, 110.9)	107.0 (105.4, 108.6)	/	0.202
Aberrant birth categories ^A^	Preterm SGA	0.3	0.2	29.707	0.000*
	Preterm AGA	3.7	2.7		
	Preterm LGA	0.8	0.3		
	Full term SGA	6.5	5.1		
	Full term AGA	63.4	71.6		
	Full term LGA	23.9	18.9		
	Post-term SGA	0.23	0.15		
	Post-term AGA	0.9	0.8		
	Post-term LGA	0.23	0.19		
Immediate family members with history of obesity related disease ^A^	Father	42.5	30.7	52.798	0.000*
	Mather	20.8	13.3	36.483	0.000*
	Paternal grandfather	42.7	38.5	6.396	0.011*
	Paternal grandmother	49.8	40.5	30.261	0.000*
	Maternal grandfather	43.2	39.8	4.153	0.042*
	Maternal grandmother	47.7	40.8	16.466	0.000*

Note. A referred toχ^2^ test, and B referred to nonparametric Mann–Whitney U test; *P <0.05, statistically significant difference. Parental educational level and career with *P*>0.1 were not listed in this table due to wide space to occupy.

**Table 3:** Multivariate non-conditional binary logistic regression analysis of risk factors associated with overweight and obesity

**Variables**	**OR**	**95% CI**	** *P* **
Aberrant birth categories			
Full term AGA	Reference group	0.45-5.82	0.459
Preterm SGA	1.621		
Preterm AGA	1.564	1.07-2.29	0.022*
Preterm LGA	2.746	1.07-7.04	0.035*
Full term SGA	1.454	1.09-1.94	0.011*
Full term LGA	1.418	1.20-1.68	0.000*
Post-term SGA	1.855	0.41-8.38	0.422
Post-term AGA	1.051	0.50- 2.21	0.896
Post-term LGA	1.297	0.30-5.64	0.728
Paternal history of obesity related disease	1.583	1.36-1.84	0.000*
Maternal history of obesity related disease	1.713	1.43-2.05	0.000*
Paternal grandparents history of obesity related disease	1.130	0.97-1.32	0.122
Maternal grandparents history of obesity related disease	1.142	0.99-1.32	0.075

Note. *P <0.05, statistically significant difference.

**Figure 1:**
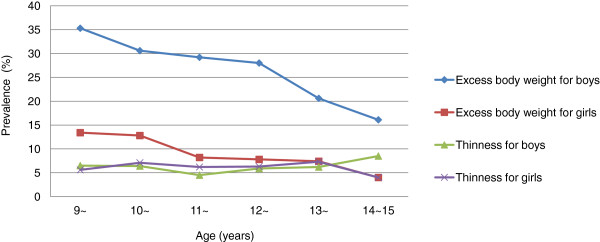
**Prevalence and tendency of thinness and excess body weight in children, stratified by age and genders.** A significant decreasing tendency with age of excess body weight prevalence was shown in both boys and girls, and the combined prevalence was obviously higher in boys than in girls (*P* <0.05). However, prevalence of thinness had significant differences neither in genders nor in ages (*P* >0.05).

The identification of risk factors is the key to prevention. Numerous risk factors for later obesity have been proposed, which generally point to nutritional imbalances in early life, fat accumulation related life styles, and socioeconomic status. In particular, asymmetric fetal development have been supposed to be potentially associated with childhood and adulthood obesity, which have recently mushroomed into a focus [7-9]. Both high and low birth weight, premature and post-mature birth, as well as small and large for gestational age, have been described as risk factors for later obesity. However, small for gestational age (SGA), preterm birth and low birth weight (LBW) were usually studied interchangeably, as if they were synonymous, as well as large for gestational age (LGA), post-term birth and high birth weight (HBW). There were sparse of studies to well elaborate the differential risk of aberrant birth categories for overweight and obesity. Additionally, family history was regarded as important risk factors [10-12]. Obesity related disease (ORD) that generally comprised of primary hypertension, T2DM, hyperlipidemia, stroke, CHD, fatty liver disease and gout belonged to one homogeneous disease with similar pathological characteristics of metabolic cardiovascular disorder. Instead of being studied separately as previous studies, they were analyzed together in our work. History of ORD of which immediate family member was the most important predictor on childhood overweight and obesity was explored. Further, there was a dearth of data on children nutritional status in city of Southwest China, but such work is rather important, especially in a large sample.

Taken together, the objectives of this study were: (i) to evaluate the nutritional status of children in a large city of Southwest China; (ii) to identify the differential impact of aberrant birth categories and family history of ORD on childhood overweight and obesity.

## Methods

### Study population

A multistage random cluster sampling method was used to select the school children aged 9–15 years. Approximately 10,000 children were selected from 9 elementary and secondary schools in 3 districts of Chengdu, and 7,194 (72.9%) who accomplished questionnaires and passed anthropometric measurements were recruited in the evaluation of nutritional status. Among them, 3,494 (48.6%) were boys and 3,700 (51.4%) were girls.

### Data collection

The survey was conducted from January to March 2011. Standardized health survey questionnaires of school children from project section of the national 11th Five-Year Plan were distributed and completed by parents or guarantees, which generally compromised of five parts: (i) baseline of children: population, gender, birth date; (ii) birth outcomes: gestational age and birth weight; (iii) family conditions including parental income and living space; (iv) immediate family members with history of ORD referring to medical standards: overweight and/or obesity, primary hypertension, T2DM, hyperlipidemia, stroke, CHD, fatty liver disease, gout, the last accurately documented height and weight. Furthermore, weight and height of children were strictly measured by specially-trained researchers. Weight was measured with the subject wearing light clothes and without shoes. Height was measured with the shoes off, feet together, and head in the horizontal plane. The weight and height were separately measured to the nearest 100g and 0.1 cm.

The study was approved by the Ethics Committees of West China Second University Hospital, Sichuan University. Prior consent for the study was obtained from the school administration and from the parents.

### Definition and category

Body mass index (BMI) was calculated as weight in kg divided by the square of height in m. Thinness, overweight, obesity and normal were defined by the new age-, sex-, specific BMI reference developed by World Health Organization (WHO) (2007) for children aged 5–19 years [13,14]. In detail, thinness was defined as BMI-for-age (Z-score) below 2 standard deviations (SD) from the median value; overweight was defined as BMI-for-age (Z-score) over 1 SD and below 2 SD; obesity was over 2 SD, both of overweight and obesity were called excess body weight; and normal was defined as BMI-for-age (Z-score) between −2 SD and 1 SD. With regard to the adults, we defined overweight with a BMI of 25.0–29.9 and obesity with a BMI ≥ 30 [15,16].

Birth outcomes including birth weight and gestational age were analyzed to identify the abnormal birth, in which preterm birth as gestation < 37 weeks, post-term birth as gestation ≥ 42 weeks, SGA with birth weight below the 10th percentile for each gestational age, LGA with birth weight above the 90th percentile for each gestational age by standards of national surveys of newborns in China. Birth outcomes were further divided into nine categories according to gestational age (preterm, full term and post-term) and birth weight for gestational age (SGA, AGA and LGA).

### Frequency matched case–control study

1,282 children who were overweight or obesity (referred as excess body weight) in total sample were all recruited to the cases. Additionally, a number of 2,586 controls were randomly selected from children with normal BMI, according to 1:2 frequency matching ratio by gender in each age group. Based on the frequency matched case–control study, the risk factors for childhood overweight and obesity were explored.

### Statistic analyses

The SPSS® statistical package, version 13.0 (SPSS, Inc., Chicago, Illinois) was applied to the statistical analyses. Continuous variables were expressed as median with 95% confidence interval, and were compared using the nonparametric Mann–Whitney U test. Categorical variables were expressed as frequencies and analyzed by means of chi-square test. In present study, the univariable analysis was first used for the initial investigation of potential risk factors, and potential covariables were selected and included in the non-conditional multivariate binary logistic regression model if they were associated with the outcome variable at *P*<0.1. The outcome variable only had two levels: children being excess body weight (overweight and obesity) or no. The final multivariate binary logistic regression model included the following covariables: aberrant birth categories (dummy variable, and full term AGA was set as the reference group), immediate family members with history of obesity related disease, parental income and living space, of which the last three were adjusted. Additionally, examined covariables not included in the final model were educational level, career. Results were presented as the adjusted odds ratio (OR) with the 95% confidence interval (CI) and corresponding *P*-values. A *P*-value of less than 0.05 was considered statistically significant.

## Results

### Prevalence of thinness, overweight and obesity

The overall prevalence of thinness, overweight and obesity for children aged 9–15 years were 6.3%, 14.5% and 3.3%, respectively. Furthermore, a total of 1,282 children (17.8%) were observed with excess body weight. The results of different age groups were presented in Table 1. As shown in Figure 1, the prevalence of excess body weight was highest in the group of 9–10 years, with a decreasing tendency with increasing age. The prevalence of overweight and obesity were much higher in boys than those in girls (21.2% vs. 8.4%, and 5.7% vs. 1.1%, *P* <0.05). However, the prevalence of thinness was similar across the age groups, as well as genders.

### Risk factors for childhood overweight and obesity

Comparisons of the study factors between two groups were presented in Table 2. The percentages of all the aberrant birth categories were separately higher in the overweight and obese children than in the controls, and were significantly related with overweight and obesity (*P* <0.001). The prevalence of preterm LGA was as high as 23.9% among children with excess body weight, in comparison with the lower 18.9% in controls. All the immediate family members including parents and grandparents of overweight and obese children were found an evident history of ORD (*P* <0.05).

The most relevant factors of overweight and obesity in the prior analyses were mainly contributed to aberrant birth and family history of obesity-related disease, and a multivariate binary logistic regression was operated to reveal the association among these predicted factors. The risk of children to become overweight and obesity was approximately three-fold higher in being born preterm LGA than full term AGA (OR 2.746, 95%CI 1.072-7.036). Additionally children born to preterm AGA, full term SGA and full term LGA were at an elevated risk of developing overweight and obesity, of which the odds ratio were 1.564 (95% CI 1.067, 2.291), 1.454 (95%CI 1.088, 1.944) and 1.418 (95%CI 1.200, 1.677), respectively. In spite of impaired birth outcomes, there were another two significant risk factors accounted for: maternal history of ORD (OR 1.713, 95%CI 1.432, 2.049) and paternal history of ORD (OR 1.583, 95%CI 1.363, 1.840) (see Table 3).

## Discussion

Obesity and overweight has become worldwide epidemic not only in adults but also in children. Given that area differences in nutritional status could not be excluded, we surveyed the nutritional status of school children aged 9–15 yrs in Chengdu, which is one of the latest cities in Southwest China. Approximately 17.8% of school children were in excess body weight, of which 14.5% were overweight and 3.3% were obesity. The combined prevalence was twice higher than that reported in the retrospective evaluation of nutritional status in children from the 2002 China National Nutrition and Health Survey (CNNHS), which applied the same reference of WHO (2007) [17]. Whereas it was still lower than the percentage of 25.7% among 10–15 yrs children in Beijing also with the same reference [18]. The prevalence of obesity in this study was a little lower than that reported among children aged 10–15 yrs in Xi`an, which was also a relatively backward city in West China [19]. Further compared to Asia Indian, Chengdu had a much lower prevalence of obesity [20]. Compared with children aged 6–12 yrs in Chengdu 3 years before, it had nearly increased 5% [21]. Trend in overweight and obesity prevalence in inland big cities of China was critically in sharp increment. Our study showed that boys were much more prevalent of overweight and obesity than girls, and the same tendency were found in other studies [18,19,22,23]. It might contribute to the different physical growth and eating behaviors between boys and girls. Girls always eat much less than boys to keep slender. And the particular pattern of pubertal development that children were more liable to rapid accumulation of body fat and bone growth at the starting of puberty, might help to explain the gradually decrement of the combined prevalence of overweight and obesity when age increased.

While overweight and obesity of children had definitely become a great public health concern, malnutrition still existed and had significant impact on the physical health of the population, especially in developing countries [1]. In present study, 6.3% of school children were thinness. There was a distinct decline from the 12-13% thinness rate of children aged 10–18 yrs from eight provinces of China in 1991–1993 [24], but a little decline from the national survey of 7.4% in 2004 using the same reference [17]. Furthermore, it was close to that of Portugal [25] and Seychelles [26], but lower than that reported in West Bengal of India [27]. Therefore, it implied that the malnutrition status was decreasing slowly in recent years in Chengdu. While we are arguing to focus on the overweight and obesity epidemic, we should still pay attention to the lasting problem of malnutrition in children in backward cities.

This study suggested that children born to preterm LGA were nearly at three times higher estimated risk to become overweight and obesity. Till now, there were few reports upon the relationship of preterm LGA with later overweight and obesity, whereas it was usually presented separately. Hediger et al. proposed that LGA infants grew much longer and heavier [28,29], and these children were suggested being prone to accumulate excess fat and become overweight or obesity [30,31]. In spite of LGA, preterm infants were also shown a higher risk of developing overweight and obesity in later life [32]. Unexpectedly, preterm infants with higher birth weight but not lower birth weight were found associated with overweight and obesity among the children. It was relatively similar to our findings. Additionally, children born to full term SGA were also found liable to develop overweight or obesity in our work. This was consistent with some previous studies, which suggested that SGA children appeared to dramatically transit toward central adiposity, mainly as a result of the rapid weight gain in later life [7,33,34]. However, we failed to demonstrate the relationship between preterm SGA birth and later overweight or obesity, which was proposed by other studies [32,35]. This might arise from the differences in study design, e.g. sample size, characteristics of objects and methodology, and further longitudinal cohort study was warranted here. Additionally these results might be weakened because of the lack of several important potential confounding factors, like dietary energy intake and physical activity.

The offspring of mothers had a history of ORD were dramatically prone to become overweight and obesity, and it was similar in children with such a paternal history, whereas at a bit lower risk. The results were scarcely reported on our inspection of other literature, and associations of family history of those ORD with childhood overweight and obesity were mostly analyzed separately. For instance, children of obese mothers showed higher risk for obesity compared with children of non-obese mothers [36,37]. Mothers who had been diagnosed with diabetes or gestational diabetes or who received diabetes treatment were recognized significantly more likely to had overweight children [12]. The reason for parental history of ORD as potential factors for childhood overweight and obesity might be mainly ascribed to the family environment and inheritance. Children`s eating patterns were substantially influenced by the caregivers, in particular mothers. The behavioral eating traits of obese parents like high-fat and/or high-calorie diet preference, excessive food intake or night snack would significantly promote the excessive weight gain of children [38]. Further, the genetic factor and newly named transgenerational epigenetic inheritance may help to explain the significant impact of parental history of ORD on the development of offspring overweight and obesity [39,40].

### Limitation of the study

Our study have a few strength including a large sample size, using recently released WHO`s growth references, and considering a novel association between the variety of distinct aberrant birth categories and childhood overweight and obesity, especially in China. However, it was designed as a cross-sectional and retrospective study, the retrospective bias was unavoidable. It has been reported that the association between birth outcome and childhood overweight or obesity may be confounded by several potential factors, such as, parental socio-economic status, family history of obesity related disease, dietary energy intake and physical activity among childhood, etc. [41,42]. In present study, parental educational level and career, which were firstly analyzed in the univariable analyses and the *P* value were all above 0.1, were not entered into the multivariate binary logistic regression. With regard to the dietary energy intake and physical activity among childhood, they weren’t available in the study, and it is a major deficiency of our study. However, these unmeasured factors are most probably not systematically related to the outcome, but may weaken the observed association. There might be several reasons. First, we surveyed a sample size as large as more than 10,000 school children, and the self-description of dietary energy intake were usually widely different although unified measurement methods were informed, according to our experience in previous studies. Second, only a little spare time except the PE classes scheduled was spent for extra physical activity, as a result from a quite hard learning task not only in school but also at home. In addition, most of school children in Chengdu had their lunch at school almost having the same dishes. Third, one recent Chinese study indicated that the relative risk of high weight-for-length/height in children aged 1–3 years associated with macrosomia was only attenuated by 6% (odds ratios from 2.33 to 2.48) after further control for postnatal illness status and feeding modalities [42]. Additionally, we had already been tracking the detailed birth information of these enrolled children in their birth hospitals, meanwhile more information about environment and life styles, as well as some laboratory and imaging index would be analyzed together in later work.

## Conclusions

Overweight and obesity was dramatically spreading among school children in Chengdu. Meanwhile, malnutrition still remained a serious problem. This unmatched nutritional status should be emphasized in relatively backward Southwest city of China. Effective prevention and intervention to childhood overweight and obesity as early as possible were imperative. Early identification of the risk factors was extremely important. So far as our study was concerned, children born of both preterm and LGA, whose parents particularly mothers had a history of ORD, should be emphatically intervened as early as possible.

## Competing interests

The authors have no competing interests to declare.

## Authors’ contributions

PL participated in the design of the study, data collection and management, data processing and statistic analysis, and the manuscript writing. YF participated in the study design, coordinated the study personnel, trained the researchers, and participated in data processing and analysis, and the manuscript writing. FX and TZH participated in the study design, data collection and data processing. YT participated in the data management, processing and analysis, and the manuscript review. SFY participated in the study design and manuscript review. MM conceived of the study, and participated in its design and supervision, and helped to draft the manuscript and review it. All authors read and approved the final manuscript.

## Pre-publication history

The pre-publication history for this paper can be accessed here:

http://www.biomedcentral.com/1471-2458/12/636/prepub
